# Administration of *Rhodiola kirilowii* Extracts during Mouse Pregnancy and Lactation Stimulates Innate but Not Adaptive Immunity of the Offspring

**DOI:** 10.1155/2017/8081642

**Published:** 2017-10-30

**Authors:** Sławomir Lewicki, Ewa Skopińska-Różewska, Aleksandra Brewczyńska, Robert Zdanowski

**Affiliations:** ^1^Department of Regenerative Medicine and Cell Biology, Military Institute of Hygiene and Epidemiology, Kozielska 4, 01-163 Warsaw, Poland; ^2^Pathomorphology Department, Center for Biostructure Research, Warsaw Medical University, Chałubińskiego 5, 02-004 Warsaw, Poland

## Abstract

The use of antibiotics during pregnancy and lactation is associated with an increased risk of developmental disorders. One of the natural medicinal plants—*Rhodiola kirilowii*, widely used as an immunostimulant in adults—might be a good alternative to antibiotic treatment. The aim of present study was to assess whether daily oral administration of 20 mg/kg of *Rhodiola kirilowii* aqueous (RKW) or 50% hydroalcoholic (RKW-A) extracts affected hematological and immunological parameters of 6-week-old mouse progeny. There was no significant change in hematological parameters of blood with the exception of hemoglobin, which was significantly higher (about 4%) in RKW group. Offspring of mothers fed *Rhodiola kirilowii* extracts had increased percentage of granulocytes and decreased percentage of lymphocytes. These changes correlated with decreased percentage of CD3^+^/CD4^+^ T-cells (RKW and RKW-A), decrease of CD8^+^ cells, and increase percentage of NK cells in RKW group. In addition, both types of *Rhodiola kirilowii* extracts stimulated granulocyte phagocytosis and increased level of respiratory burst. In conclusion, the long-term supplementation of mouse mothers during pregnancy and lactation with RKW or RKW-A extracts affects the immune system of their progeny. These results should be taken into consideration before administration of *Rhodiola kirilowii* to pregnant and lactating women.

## 1. Introduction

Treatment of illnesses during pregnancy and in early infancy can be problematic because of its potential effects on development of infant immune system [1]. Vast majority of infections are treated with antibiotics that cause plethora of side effects. FDA (Food and Drug Administration) of the U.S. Department of Health and Human Services created a list of antibiotics, divided into 5 groups (A, B, C, D, and X) depending on the potential risk of side effects. Only antibiotics from group A are considered as being safe to use during pregnancy and lactation [2]. The validity of such a classification was confirmed in studies conducted on mouse model. Using experimental model of pregnant mice, Skopińska-Różewska and collaborators showed that prenatal exposure to antibiotics modifies the immune system in progeny [3, 4]. Moreover, the offspring of mothers treated during pregnancy with antibiotics from penicillin and cephalosporin group (group B) showed an altered reactivity of the immune system manifested by decreased intensity of cellular response and increased intensity of humoral immune response to pathogens. In addition, a prenatal and postnatal exposure to antibiotics is associated with an increased risk of asthma and obesity in childhood [5].

The overuse of antibiotics not only in medicine but also in food industry causes increasing antibiotic resistance and other side effects [6–8]. Thus, there is an increasing demand for a natural antimicrobial and immunostimulatory medications, which could be safely administered during pregnancy; the herbs from the *Rhodiola* genus seem to be an excellent candidate.

*Rhodiola* genus from *Crassulaceae* family consists of more than 200 species out of which over 20 species have medicinal properties. They were used in traditional medicine of Asia and Europe as tonic, adaptogen, antidepressant, and anti-inflammatory drugs, and for these properties, they are being tested recently in some clinical trials [9–11]. Clinical trials showed lack of adverse effects and interactions between *Rhodiola* extracts and other drugs. Plants from *Rhodiola* species are known not only as a diet supplement that supports mental and physical performance of the body but also for the beneficial antitumor, antimicrobial, and immunomodulatory activity [12, 13]. Importantly, *Rhodiola* has antiviral and antimicrobial properties against hepatitis C virus (HCV) and *Mycobacterium tuberculosis* [14, 15]. Currently, several novel clinical therapies based on the abilities of *Rhodiola* extracts to stimulate the immune system are under development [16].

All these properties make *Rhodiola* an attractive source for producing various medications. Biological activity of *Rhodiola* spp. depends on the phytochemicals such as polyphenols, phytosterols, caretonoids, saponines, and alkaloids. Using mouse model, we showed that *Rhodiola kirilowii*, *R*. *rosea*, and *R*. *quadrifida* extracts stimulated granulocyte and lymphocyte activity. Feeding mice with *Rhodiola* extracts for 7 days lowered intensity of *Pseudomonas aeruginosa* infection, increased blood leukocyte number, and had modulatory effect on their metabolic activity [17–22].

The aim of the present study was to investigate if and how 50% hydroalcoholic (RKW-A) and aqueous (RKW) extracts of underground (roots and rhizomes) parts of *Rhodiola kirilowii* administrated to pregnant and lactating mice stimulate innate immunity of the offspring.

## 2. Material and Methods

### 2.1. Plant Cultivation

*Rhodiola kirilowii* (Crassulaceae) plants were collected from the Institute of Natural Fibers and Medical Plants (Poznań, Poland) field cultivations of herbal plants. The taxonomic status of plant was confirmed with *Flora of the Soviet Union* (Vol. 9, 1939) and *Flora of China* (Vol. 8, 2001). A voucher specimen is kept in the herbarium of the Department of Botany, Breeding and Agriculture in Plewiska near Poznań.

### 2.2. Preparation of Extracts

Extracts have been prepared as previously described [23]. Briefly, for RKW extract production, the finely powdered *R. kirilowii* roots were extracted twice with water (first for 2 h and then for 1 h) in a raw material to solvent ratio of 1 : 5, at temperature between 40 and 45°C. Supernatants from each extraction were mixed together, centrifuged (15 min, room temperature, 2000 ×g), and lyophilized. RKW extract contained about 14 *μ*g of analyzed polyphenols (kaempferol, epicatechin, quercetin, (+)-catechin, salidroside, fisetin, naringenin, luteoli, p-coumaric acid, ellagic acid, epigallocatechin, ferulic acid, chlorogenic acid, epicatechin gallate, and epigallocatechin gallate) per mg of dry extract. For RKW-A extract production, the finely powdered *R. kirilowii* roots were extracted with a 50% ethanol, in a raw material to solvent ratio of 1 : 10 using the percolation method. The percolates were lyophilized following distillation at 40–45°C. RKW-A extract contained about 21 *μ*g of analyzed polyphenols.

Dry extract ratio (g of medical herb to g final extract) was 5.09 : 1 for RKW and 3.27 : 1 for RKW-A. Extracts were stored at −70°C until further use.

### 2.3. Animals

All animal experiments were conducted according to the Polish regulation and standards of the wellness of laboratory animals. All experiments were accepted by and conducted according to the ethical guidance of Local Bioethical Committee (permission 73/2011).

Experiments were performed on 6-week-old progeny of 70 adult inbred female BALB/c mice (8-9 weeks old; ~20 g weight; purchased from Mossakowski Medical Research Centre Polish Academy of Sciences, Warsaw, Poland), which were mated with adult males of the same strain. After confirmation of pregnancy (the presence of copulatory plug), Balb/c females were fed daily (up to the 28th day after delivery) with lyophilized RKW or RKW-A extracts (20 mg of extract/kg of body weight) dissolved in sterile water. The control group received sterile water only. To avoid stress related to gavage feeding and handling that can lead to miscarriage, 20 microliters of tested substance dissolved in sterile water was placed on one corn chip and served to the mouse in a petri dish. Female and male progenies were housed separately.

Mice were maintained under conventional conditions (22.5–23°C, relative humidity 50–70%, 12 h day/night cycle) with free access to breeding rodent feed (Labofeed H, Wytwórnia Pasz “Morawski”) and water. Pups were withdrawn from mothers 24 days after delivery.

### 2.4. Blood Isolation

Mice were anesthetized (intraperitoneal injection of ketamine (120 mg/kg) and xylazine (12 mg/kg); Polypharm S.A., Warsaw, Poland), and retro-orbital blood was collected in the EDTA-containing tubes (for further hematological and immunological analysis) and heparin-containing tubes (for further phagocytosis and oxidative burst analysis).

### 2.5. Hematological Analysis

Blood (50 *μ*l from EDTA tubes) was analyzed in a hematological analyzer (Exigo veterinary hematological system; Boule Medical AB, Stockholm, Sweden). The following parameters were evaluated: WBC (white blood cells), RBC (red blood cells), HGB (hemoglobin), HCT (hematocrit), MCV (mean corpuscular volume), MCH (mean corpuscular hemoglobin), MCHC (mean corpuscular hemoglobin concentration), RDW% (red cell distribution width (%)), RDW-a (red cell distribution width absolute), PLT (platelets), and MPV (mean platelet volume). Additionally, WBC number and cell percentage from flow cytometry analysis were used to calculate a total number of lymphocytes, lymphocyte subpopulations, monocytes, and granulocytes per *μ*l of blood. The results are presented as the mean ± standard error.

### 2.6. Determination of White Blood Cell Phenotype

Determination of WBC phenotype was performed as described previously for splenocyte phenotyping [24]. Briefly, the cells in whole blood (100 *μ*l from EDTA tubes) were labeled with the following fluorochrome-conjugated anti-mouse monoclonal antibodies: Mouse T lymphocyte Subset Antibody Cocktail with Isotype Control (hamster anti-mouse cluster of differentiation (CD) 3e, rat anti-mouse PE CD4, and rat anti-mouse CD8a; cat. number 558431), Mouse B Lymphocyte Activation Antibody Cocktail with Isotype Control (rat anti-mouse CD25, hamster anti-mouse CD69, and rat anti-mouse CD19), and rat anti-mouse CD335 (natural killer cell p46-related protein all purchased from BD Biosciences, Warsaw, Poland). Staining (20 min incubation at room temperature) was performed according to the manufacturer's instructions. Subsequently, the red blood cells were lysed (10 min, Lysing Solution 10x Concentrate; BD Biosciences) and remaining cells were washed twice with PBS. Phenotypic analysis was performed by flow cytometry (FACSCalibur; BD Biosciences). Additional phenotypic determination of WBC population (lymphocytes, monocytes, and granulocytes) was made using FSC/SSC parameters. The results are presented as the mean % ± standard error of the mean of WBC (for lymphocytes, monocytes, and granulocytes) or lymphocytes (for CD3, CD4, CD8, CD19, and CD335 analysis) and also as mean ± standard error of cell number in liter of blood.

### 2.7. Treg Analysis

Mouse Treg cell evaluation was performed analogously as previously described for Treg cells [25]. Briefly, cells in whole blood samples (100 *μ*l) were immunostained with 20 *μ*l of primary antibodies CD4-PerCP and CD25-APC (extracellular staining, BD Biosciences, Poland) or an appropriate isotype control for 20 minutes at room temperature. Subsequently, erythrocytes were lysed in BD FACS Lysing Solution for 10 minutes at room temperature. Remaining immune cells were fixed, permeabilized (fixation/permeabilization buffer) in accordance with the manufacturer's protocol (BD Pharmingen, Poland), and stained with 20 *μ*l of FoxP3 PE or isotype IgG1 kappa PE antibody (45 min/RT in the dark). Afterwards, the cells were washed twice with PBS and fixed in 300 *μ*l of 1% PFA in PBS solution. The cells were counted by flow cytometry—10000 counts of CD4-PerCP-positive cells stopped the acquisition. Evaluation of CD4^+^/CD25^+^ cells or Treg cells (CD4^+^/CD25^+^ and FoxP3^+^) was performed using CellQuest Pro software (BD). The results are presented as the mean percentage of CD4^+^ cells ± standard error of the mean.

### 2.8. Phagocytosis

Blood cell phagocytosis was assessed by PHAGOTEST™ (BD Biosciences, Warsaw, Poland) according to the manufacturer's protocol. 100 *μ*l of whole blood (collected in heparin tube) was used per test. Blood was incubated for10 min. With opsonized GFP-stained *E. coli* in control (0°C) and experimental (37°C) conditions, then quenching solution was added, and red blood cells were lysed in lysing solution. Level of phagocytosis (in granulocytes or monocytes) was measured by flow cytometry (FACSCalibur). The results are presented as the mean % of cells (granulocytes or monocytes) containing phagocytized *E. coli* ± standard error of the mean.

### 2.9. Oxidative Burst

Quantitative determination of leukocyte oxidative burst was performed in heparinized whole blood using PHAGOBURST™ assay (BD Biosciences, Warsaw, Poland) according to the manufacturer's protocol, with our own modification. Standard kit contains unlabeled opsonized *E. coli* bacteria as particulate stimulus, the protein kinase C ligand phorbol 12-myristate 13-acetate (PMA) as high stimulus, and the chemotactic peptide N-formyl-Met-Leu-Phe (fMLP) as low physiological stimulus. We modified protocol by replacing PMA with the zymosan (20 *μ*g/ml). Oxidative burst analysis was performed by flow cytometry (FACSCalibur). The results are presented as the mean % of cells (granulocytes or monocytes) with oxidative burst ± standard error of the mean.

### 2.10. Statistical Analysis

Statistical evaluation of the results obtained, from the control and experimental groups, was performed using unpaired *t*-tests and one- or two-way analysis of the variance, followed by the Tukey test or Bonferroni correction (in the case of a normal distribution) or nonparametric Kruskal-Wallis and Mann–Whitney *U* tests (in the case of abnormal distribution). Assessment of the distribution of the data was evaluated using the Shapiro-Wilk test. GraphPad Prism software was used to carry out these tests (version 5; GraphPad Software Inc., La Jolla, CA, USA). *P* < 0.05 was considered as statistically significant.

## 3. Results

### 3.1. Hematological Analysis

There was no significant difference in RBC, HCT, MCH, MCHC, RDW%, RDWa, PLT, and MPV parameters between control and experimental groups. The offspring of mice fed during pregnancy and lactation with water extract of *Rhodiola kirilowii* exhibited slightly higher (about 4%, *P* = 0.0325, statistically significant) concentration of hemoglobin in blood in comparison to control group. There was also a slight but statistically significant (*P* = 0.0354) difference between RKW and RKW-A group in mean corpuscular volume. The results of hematological analysis are presented in Table 1.

### 3.2. WBC Analysis

Water and hydroalcoholic extract of *Rhodiola kirilowii* did not affect the total number of WBC populations (lymphocytes, monocytes, and granulocytes) or percentage of monocytes in blood when compared to control group. Both extracts of *Rhodiola kirilowii* decreased proportion of lymphocytes and increased proportion of granulocytes. However, statistically significant differences (approximately 7% for lymphocytes (*P* = 0.0288) and 12% for granulocytes (*P* = 0.0243)) were observed for water extract of *Rhodiola kirilowii*. Described results are presented in Table 2.

### 3.3. Lymphocyte Phenotyping

**Figure 1:**
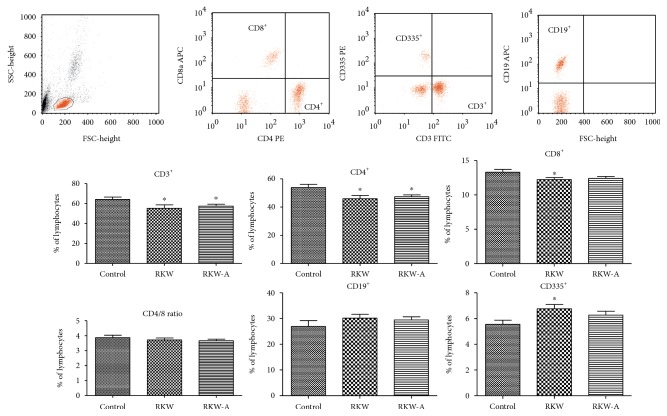
Blood lymphocyte phenotype analysis (mean % ± SEM). Analysis was performed in the offspring of mothers fed during pregnancy and lactation with water (RKW) or hydroalcoholic (RKW-A) extract of *Rhodiola kirilowii*. ^∗^*P* < 0.05.

The offspring of mice that received *Rhodiola kirilowii* extracts during pregnancy and nursing period exhibited significantly lower percentage of CD3^+^ (approximately RKW-14%, *P* = 0.0434; RKW-A-10%, *P* = 0.0337) and CD4^+^ cells (15%, *P* = 0.0184 and 13%, *P* = 0.0116, resp.) in comparison to control group. Feeding with RKW extract caused statistically significant decrease in percentage of CD8-positive cells (*P* = 0.0342) and increase in percentage of CD335-positive NK cells (*P* = 0.0134) in comparison to control. There was no significant change in B-cell percentage between all studied groups (Figure 1).

**Figure 2:**
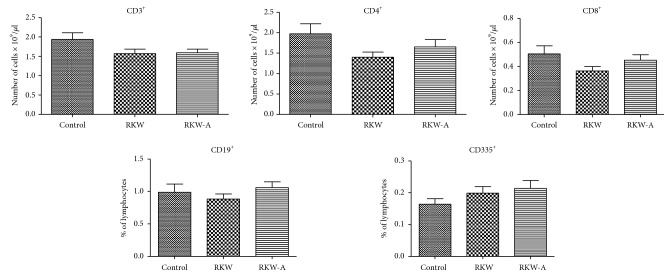
Blood lymphocyte phenotype analysis (cell number per *μ*l). Analysis was performed in the offspring of mothers fed during pregnancy and lactation with water (RKW) or hydroalcoholic (RKW-A) extract of *Rhodiola kirilowii*.

There were some slight changes in the total number of lymphocytes in each studied subpopulation; however, the differences between control and extract-fed groups were not statistically significant (Figure 2).

### 3.4. Treg Analysis

**Figure 3:**
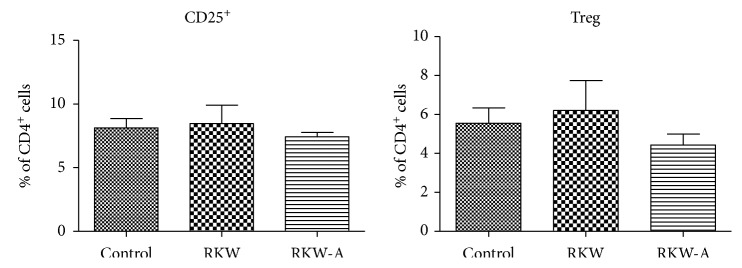
Treg cells in peripheral blood. Analysis was performed in the offspring of mothers fed during pregnancy and lactation with water (RKW) or hydroalcoholic (RKW-A) extract of *Rhodiola kirilowii.*

There was no statistically significant difference in the percentage of CD4^+^/CD25^+^ and Treg cells between examined groups (Figure 3).

### 3.5. Phagocytosis

**Figure 4:**
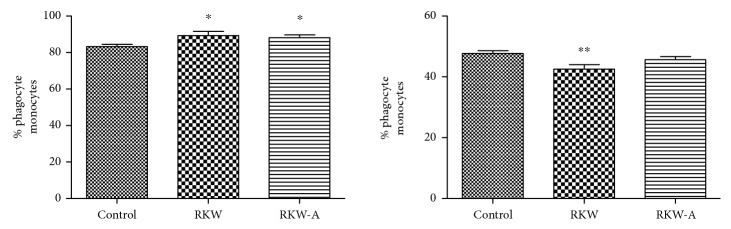
Blood cell phagocytosis. Analysis was performed in the offspring of mothers fed during pregnancy and lactation with water (RKW) or hydroalcoholic (RKW-A) extract of *Rhodiola kirilowii*. ^∗^*P* < 0.05; ^∗∗^*P* < 0.01.

Both extracts of *Rhodiola kirilowii* significantly stimulated granulocyte phagocytosis as measured by the number of cells containing opsonized *E. coli*, when compared to control group. The stimulation was approximately 7% higher in RKW group (*P* = 0.0311) and 6% in RKW-A group (*P* = 0.0370). Interestingly, monocytes isolated from blood of the offspring, which mothers were fed during pregnancy and nursing with water extract of *Rhodiola kirilowii*, exhibited significantly lower level of monocyte phagocytosis (about 12%, *P* = 0.0075). Level of monocyte phagocytosis in RKW-A group remained unaffected (Figure 4).

### 3.6. Oxidative Burst

**Figure 5:**
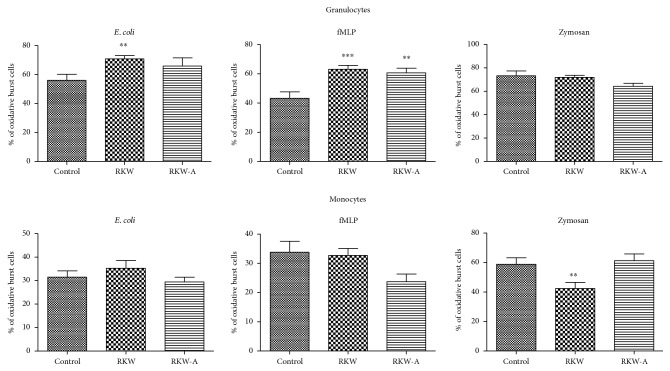
Oxidative burst. Analysis was performed in the offspring of mothers fed during pregnancy and lactation with water (RKW) or hydroalcoholic (RKW-A) extract of *Rhodiola kirilowii*. ^∗∗^*P* < 0.01; ^∗∗∗^*P* < 0.001.

Similar to phagocytosis, extracts of *Rhodiola kirilowii* administrated to pregnant and lactating mice increased level of respiratory burst stimulated by exposure to *E. coli* or fMLP. The granulocytes from RKW group exposed to *E. coli* (strong stimulant of oxidative burst) showed approximately 26% enhancement (*P* = 0.0021) of oxidative burst when compared to control. Addition of low physiological stimulant, fMLP caused significant increase of granulocyte respiratory burst in both RKW and RKW-A groups (46% with *P* = 0.0010 and 40% with *P* = 0.0066, resp.). There was no significant change after zymosan (all groups) and *E. coli* (RKW-A group) exposure (Figure 5).

There was also no significant difference between control and *Rhodiola kirilowii* groups in monocyte oxidative burst after *E. coli* and fMLP stimulation. The statistically significant decrease of oxidative burst occurred only after zymosan stimulation and only in RKW (around 28%, *P* = 0.0098) group. There was no difference in RKW-A group (Figure 5).

## 4. Discussion

The immune system of fetus develops slowly during pregnancy and rapidly during the first period after birth [26]. Proper functioning of the immune system is essential for defense against pathogens and regulation of homeostasis. Our previous experiments in mouse model showed that *Rhodiola kirilowii* extract affects certain parameters of pregnancy such as litter size and number of females without progeny [23, 27].

In this study, we showed that the *Rhodiola kirilowii* extracts administrated to the mouse mothers during lactation and nursing did not affect the hematological parameters. The only exception was a slight but statistically significant increase in the concentration of hemoglobin in the group supplemented with an aqueous extract of *Rhodiola kirilowii*. Similar results were obtained by Gupta et al. [28] with another *Rhodiola* species. In these studies, rats treated with *Rhodiola imbricate* did not show significant changes in the blood. Also, Senthilkumar et al. [29] had not observed any significant differences in blood parameters of Wistar rats after administration of the acetone extract of *Rhodiola imbricata* (200 and 400 mg/kg). Moreover, *Rhodiola imbricata* supplementation showed hepatoprotective properties against paracetamol-induced liver toxicity.

There are many factors, which are able to modify (increase or decrease) the population of WBC cells. Such biologically active compounds are probably present in water extract and hydroalcoholic extract of *Rhodiola kirilowii*. In our previous study, we showed that both extracts of *Rhodiola kirilowii* contain kaempferol, epicatechin quercetin, (+)-catechin, salidroside, fisetin, naringenin, luteolin, p-coumaric acid, ellagic, acid epigallocatechin, ferulic and chlorogenic acid, epicatechin gallate, and epigallocatechin gallate [27]. Some of these compounds were also found in sera of mouse mothers [30]. It is known that some of biological compounds found in *Rhodiola kirilowii* may affect the number, percentage distribution, and activity of WBC populations. For example, the salidroside modulates mouse inflammatory responses, both in the number of immunological cells and in secretion of inflammatory cytokines [31]. It also positively affects bone marrow (BM) function by modulating the number of peripheral white blood cells in bone marrow-depressed mice [32].

We showed here that the offspring of mice that received *Rhodiola kirilowii* extracts during pregnancy and nursing period exhibited statistically significant lower percentage of CD3^+^ T-cells and decrease of CD4^+^ T-cells, in comparison to control group. CD4^+^ T-cells are important for adaptive immunity response [33]. These cells recognize MHC class II protein complex and are involved in the induction and the restraint of most immunological functions. A deficiency of CD4^+^ T-cells causes significant attenuation of adaptive response. This is especially visible in HIV patients where reduction of CD4^+^ T-cells causes immunological failure [34]. To confirm this conjectures, in our previous studies [24], we checked the functionality of the components of adaptive response in the progeny after *Rhodiola* extract administration. Spleen cell number and spleen cell phenotype (CD3^+^, CD4^+^, CD8^+^, and CD19^+^) were not affected after RKW treatment. Also, spleen cell proliferation (after lipopolysaccharide and phytohaemagglutinin) was not affected; however, we noted significant reduction of spleen cell proliferation after concanavalin A stimulation in RKW group, which is somewhat worrying. Reduction in the percentage of CD4^+^ cells observed in previous work was also not associated with the significant reduction in the total number of CD4^+^ cells. CD4^+^ T-cells cooperate with B-cells and together promote strength and duration of adaptive immune response. Here, we showed that there was no significant difference in B-cell percentage and number between *Rhodiola*-treated and control groups. All these data indicated that RKW extract did not have negative effect on the adaptive functionality. Administration of SRBC to the progeny which mothers were fed with RKW extracts during pregnancy and nursing did not influence antibody production [24]. In contrast, the hydroalcoholic extract of *Rhodiola kirilowii* significantly decreased the level of antibodies in serum (SRBC test) and significantly reduced spleen cell proliferation. This suggests that about 33% increase of polyphenols in mothers' diet (differences between RKW and RKW-A) significantly affects adaptive immune response.

Similar situation (significantly lower percentage of CD8^+^ T-cells in the blood) was observed in RKW group. Decreased level of both T-cell populations suggests their faulty production in bone marrow or faulty development in the thymus. However, the results of our previous studies indicated that feeding pregnant and lactating mothers with water or hydroalcoholic extract of *Rhodiola kirilowii* decreased thymus T lymphocyte apoptosis and did not significantly affect IL-7 expression in progeny's thymocytes [35]. Similar results were reported by Liu et al. [36] in the model of septic rats after administration of *Rhodiola rosea* (50 mg/kg body weight). These changes correlated with downregulation of tumor necrosis factor-*α*-induced protein 8-like 2. Taken together, these results indicate that the extracts from *Rhodiola* help to preserve thymus function in the progeny of treated animals. We believe that a decreased level of CD8^+^ T-cells in the blood may be a consequence of the increased mobility of these cells. Our previous studies showed significantly higher number of CD8^+^ T-cells in the spleen of the progeny whose mothers were fed with *Rhodiola kirilowii* extracts [37]. Moreover, CD8^+^ T-positive cells were found in the nontypical location within the spleen, that is, not only in the PALS but also in the B-cell follicle and in the red pulp.

The lack of effect of RKW extracts on the adaptive immunity is also confirmed by the results of Treg cell analysis. Our present study showed that there was no significant difference in the total number and percentage of regulatory T-cells (Treg) between control and *Rhodiola*-treated groups. Also, Xu et al. [38] did not observe significant changes in the ratio of circulating Tregs or Treg cell differentiation in the mice treated with *Rhodiola rosea*. These results should be seen as positive outcome of the treatment, because changes in the number and percentage of Tregs may be one of the reasons for the development of autoimmune, allergic, or malignant diseases [39].

Another positive outcome of the *Rhodiola* treatment is visible in the behavior of NK cells. We observed significant increase of NK cell percentage in the blood of mice whose mothers were fed during pregnancy and lactation with water extract of *Rhodiola kirilowii*. The NK cells exhibit cytotoxic properties and are the part of the innate immune system. The NK cells survey the body for aberrant expression of MHC class I molecules and stress markers on the autologous cells [40]. Increased number and activity of NK cells are especially desirable for negative regulation of growth and metastasis of tumors. Lee et al. [41] showed that NK cells isolated from human blood reduce systemic metastasis and inhibit development of glioblastoma cells in nude mouse. Also, Diwaker et al. [42] noted increased number of NK cells after *Rhodiola* treatment in dengue-virus-infected human PBMCs.

Phagocytosis is a major process in which granulocytes and monocytes eliminate pathogens [43]. Several studies showed that the granulocytes exhibit higher level of phagocytosis than monocytes [44, 45]. Filias et al. [46] showed that phagocytotic ability of granulocytes and monocytes in the neonates is fully functional few days after birth. Although the standard isolation procedures do not affect phagocytosis of *Escherichia coli* bacteria [47], the isolation procedures may affect the granulocyte respiratory burst but only after anti-CD15 antibody-conjugated microbead isolation (positive selection).

We observed an increased level of phagocytic activity in granulocytes from *Rhodiola* extract group. Additionally, there were changes in oxidative burst after strong (*E. coli*) and weak (fMLP) stimulation. RKW groups showed higher stimulation of phagocytosis and oxidative burst than RKW-A groups. There is strong evidence that herbs from *Rhodiola* species stimulate innate immunity by enhancement of phagocytosis. These effects were found in several mammalian species (mice, rats, and pigs) after administration of *Rhodiola quadrifida* or *Rhodiola rosea* [20, 21]. Administration of *Rhodiola kirilowii* to the mouse mothers during pregnancy and nursing significantly improves activity of innate immunity cells of their offspring. These are very promising results; however, further studies are needed to elucidate the mechanisms of *Rhodiola* action in pregnancy and lactation.

Cytokines are hormone-like messengers regulating development and functionality of both adaptive and innate immune responses [48, 49]. There are several studies describing the effects of *Rhodiola* spp. on the cytokine secretion. Lin et al. [50] showed increased secretion of both Th1- and Th2-pattern cytokines in a dose- and time-dependent manner after *Rhodiola rosea* treatment. The *Rhodiola rosea* supplementation enhanced production of interferon (IFN) *β* and other cytokines, including IL-1*β*, TNF-*α*, IL-6, and IL-8, in the monocytes infected with dengue virus [42]. We also checked the expression of cytokines in the serum of the progeny which mothers were fed during pregnancy and lactation with the RK extracts. The RKW and RKW-A extracts supplementation did not change the expression of IFN *γ*, IL-2, IL-4 or IL-6 in progeny sera compared with the control. However, TNF-*α* and IL-10 expression was higher in the progeny of mice fed with RKW-A extract [24]. The RKW administration lowered the concentration of interleukin-17a. It is known that IL-17a induces and mediates the proinflammatory responses and promotes production of IL-6, TNF-*α*, GCF, GM-CSF, IL-1*β*, TGF-*β*, and IL-8 [51, 52].

Our present and previously published studies tried to assess, which one of the two extracts, water (RKW) or hydroalcoholic (RKW-A) of *Rhodiola kirilowii* when given during pregnancy and lactation period that are less detrimental to the health of the offspring. We showed that the water extract (RKW) is less detrimental to the health of the offspring. This conclusion is based on the following data:

- The offspring of mothers fed during pregnancy and nursing period with water extract (RKW) of *Rhodiola kirilowii* exhibits stimulation of innate immune response (granulocytes and NK cells) and slight decrease of monocyte activity (present work), without changes in adaptive immune response (SRBC blood test). In contrast, feeding with RKW-A impaired adaptive response [24].
- There were no significant differences in spleen cell response after lipopolysaccharide and phytohaemagglutinin, and there was a decreased response after concanavalin A stimulation in RKW group. In contrast, feeding with RKW-A diminished response to all these stimulants [24].
- Both extracts protected thymus cells from apoptosis [35].
- The offspring of RKW-fed group, in contrast to RKW-A-fed group, had no significant differences in kidney structure and function [53]. In addition, mice from RKW-A group had higher level of angiogenic factors (VEGF and bFGF) in serum [30].
- There were no neonatal deaths in RKW and control groups and several neonatal deaths in RKW-A group [23].

Taking together, these results suggest that water extract of *Rhodiola kirilowii* is safer to use during pregnancy and lactation.

## 5. Conclusions

Long-term supplementation of mouse mothers during pregnancy and lactation with water extract or hydroalcoholic extract of *Rhodiola kirilowii* affects some parameters of the immune system of their progeny. These treatments increase the number and activity of innate immune cells in blood and slightly decrease the percentage of T-cells (mainly CD4^+^). The results obtained in the present work provide evidence that plant-derived biologically active compounds administrated to the mothers during pregnancy and nursing period may significantly and permanently affect the immune response of their offspring. These results can be useful for creation of guidelines for pregnant and lactating women's diet.

## Figures and Tables

**Table 1:** Blood hematological analysis. Analysis was performed in the offspring of mothers fed during pregnancy and lactation with water (RKW) or hydroalcoholic (RKW-A) extract of *Rhodiola kirilowii.* Bold font indicates statistically significant differences in comparison to control group (*P* < 0.05).

Parameters	Unit	Control		RKW		RKW-A	
		Mean	SEM	Mean	SEM	Mean	SEM
RBC	×10^12^/l	10.3	0.1	10.5	0.1	10.3	0.1
HGB	g/dl	15.4	0.2	**16.0**	0.2	15.7	0.2
HCT	%	55.8	0.8	57.1	0.8	56.0	0.7
MCV	fl	54.2	0.3	54.1	0.3	54.4	0.3
MCH	pg	15.3	0.1	15.4	0.1	15.3	0.1
MCHC	g/dl	28.3	0.2	28.5	0.1	28.1	0.1
RDW	%	20.6	0.4	20.5	0.6	20.4	0.3
RDWa	fl	39.1	0.5	38.4	0.6	39.1	0.3
PLT	×10^9^/l	510.1	47.8	521.6	62.2	487.2	32.9
MPV	fl	5.8	0.2	5.9	0.1	6.1	0.2

**Table 2:** Blood WBC analysis. Analysis was performed in the offspring of mothers fed during pregnancy and lactation with water (RKW) or hydroalcoholic (RKW-A) extract of *Rhodiola kirilowii*. Bold font indicates statistically significant differences in comparison to control group (*P* < 0.05).

Parameters	Unit	Control		RKW		RKW-A	
		Mean	SEM	Mean	SEM	Mean	SEM
WBC	×10^9^/l	5.49	0.57	4.66	0.30	5.36	0.44
Lymphocytes	×10^9^/l	3.70	0.37	3.00	0.21	3.51	0.33
	%	68.62	1.63	**64.02**	1.06	64.17	1.64
Monocytes	×10^9^/l	0.37	0.05	0.27	0.03	0.32	0.03
	%	6.60	0.62	5.71	0.37	5.97	0.43
Granulocytes	×10^9^/l	1.00	0.14	0.98	0.07	0.99	0.07
	%	17.82	1.10	**21.49**	1.11	19.91	1.66
